# Adapting to living with systemic autoimmune rheumatic diseases; a qualitative exploration of patient and clinician perspectives

**DOI:** 10.1007/s00296-025-06033-9

**Published:** 2025-12-15

**Authors:** Alice Tunks, Martha Piper, Sean Humfrey, Ellie Dalby, Lucy Calderwood, Kaira Naidu, Sydnae Taylor, Rosia Xiaoke Li, Shihab Ahmed, Wendy Diment, Michael Bosley, Miranda Van Emmenis, Felix Naughton, Sam Norton, Mandy Cousins, David D’Cruz, Melanie Sloan

**Affiliations:** 1 Long Term Conditions Group, Department of Public Health and Primary Care Unit, University of Cambridge, Cambridge Biomedical Campus, Forvie Site, Cambridge, CB2 0SR UK; 2 Devizes, UK; 3 London, UK; 4 Clinical School, University of Cambridge, Cambridge, UK; 5 Faculty of Medicine and Health Sciences, University of East Anglia, Norwich, UK; 6 King’s College London, London, UK; 7 The Louise Coote Lupus Unit, Guy’s and St Thomas’ Hospitals NHS Foundation Trust, London, UK

**Keywords:** Systemic autoimmune rheumatic disease, Qualitative, Adapt, Cope

## Abstract

Systemic autoimmune rheumatic diseases (SARDs) have widespread symptoms and negatively impact lives in multiple domains. This study explores self-management and coping strategies, and patient and clinician perspectives on adapting to living with a SARD. Semi-structured, in-depth interviews were conducted with patients and clinicians. Patients with SARDs (n = 31) were asked about how they adapted to, and whether they had accepted living with a SARD. Clinicians (n = 28) were asked about their experiences of patients adapting to living with a SARD and their involvement in this process. Data were analysed using reflexive thematic analysis within a critical realist framework by a multidisciplinary team including patients as equal members of the research team. Three themes were identified. The first theme identified adapting as a non-linear, ever-shifting journey. For many, this involved grief for their pre-disease identity and life, which for some resolved into acceptance and finding contentment. The second theme explored with patients and clinicians the various methods of identifying and implementing adaptations, and how adaptations often came with social and financial costs. The final theme considered the benefits of holistic and psychological support, and the limited current provision by healthcare services. Clinicians were largely very empathetic and cited lack of time and training as reasons for difficulty in addressing adaptation. Adapting to living with a SARD is highly individualised. To adapt, it is crucial to find tailored adaptations and come to terms with the unpredictable, non-linear aspects of SARDs and subsequently acceptance. As such, finding support integrating psychological support into rheumatology care. Future research should provide evidence for tailored therapeutic interventions for SARD populations.

**Supplementary Information:**

The online version contains supplementary material available at 10.1007/s00296-025-06033-9.

## Introduction

Systemic autoimmune rheumatic diseases (SARDs) are chronic conditions. In addition to their potential for organ damage and increased mortality [1, 2], fatigue, pain and mood changes are particularly common [3–5] and can greatly limit quality of life [6, 7]. Treatment can alleviate symptoms and remission can occur, however, as they are lifelong conditions, adaptations are usually necessary [8, 9]. Adaptation is defined here as processes to accommodate the impact SARDs have on one’s life. Adaptations are not treatments or solutions to SARDs, but patient-led initiatives to improve life with disease, including both practical and psychological measures. In our exploratory work, acceptance was identified as a contentious word. It is defined here as an ongoing process of recognising and coming to terms with the long-term nature and impact SARDs have on one’s life. We see acceptance as not a state which can be obtained, but a process to be engaged with.

Self-management strategies, such as problem solving and goal setting, have been recommended for patients with rheumatoid arthritis (RA) [10] and there is moderate evidence for educational, psychosocial, goal setting and response training interventions to promote adapting in inflammatory arthritis patients [11]. Previous research has defined self-management of chronic conditions to include factors such as the patient taking responsibility for the care process and being open to a reciprocal partnership with healthcare professionals [12]. Rather than using the term self-management, which implies the responsibility is solely on the patient, we use the term adapting.

Coping strategies can help people adapt to their conditions and can be categorised into emotion- and problem-focused styles. Patients with systemic lupus erythematosus (SLE) are reported to have significantly higher scores in restraint coping (limiting or controlling behaviours), emotional regulation and a greater tendency to focus on venting emotions and using problem solving strategies, compared to controls [13, 14]. Similarly, patients with RA often use active coping, planning and acceptance strategies [15]. Higher problem-focused coping has been found to correlate with lower depression and distress in autoimmune disease patients [16]. Although it is important to consider that coping strategies are context-dependent rather than inherently adaptive or maladaptive; active strategies can enhance quality of life (QoL), while passive and avoidant ones usually do not [17].

Knowledge can be used as a coping strategy. Patients report that information and knowledge about SARDs, including of triggers and flares, is an important tool to cope [18–20]. In Sjögren’s patients, coping has been found to involve three components: personalisation (trial-and-error symptom management), attitude, and knowledge [20]. Personalisation and adaptation being a dynamic process in response to changing symptoms, has been identified in other literature [14]. Subsequently, mental flexibility, identified as part of the self-regulation process, helps patients adapt to the disease [21].

Understanding the nature of patients’ adaptation is key, as research suggests it is related to disease outcomes and mental wellbeing [22]. Previous research has touched on adapting to living with SARDs when investigating patients’ experiences [18, 23]. Research has also investigated specific coping mechanisms, and/or how patients adapt to live with specific SARDs [14, 19, 24]. However, no previous research has focused on patients’ experiences of adapting across SARDs or has been patient-led for what adaptations are focused on. This study was co-produced with people living with SARDs and a multi-disciplinary team. We aimed to investigate the views and experiences of SARD patients and clinicians in terms of beliefs and mechanisms of adapting to living with SARD(s).

## Method

### Design

Qualitative data included interviews and responses to open-ended survey questions about adapting on the INSPIRE survey (32).

### Patient and Public Involvement

Patients and clinicians were involved throughout every stage of the research as equal members of the research team. This included in driving the research agenda, revising the topic guide to ensure questions were appropriately asked and certain topics were covered, and in the development and revision of themes and subthemes.

### Materials

Demographic data and open-ended responses were collected in the INSPIRE survey. The interview topic guide was developed based on the teams’ varied and extensive experience and guided by previous literature. Topics included whether and how patients adapted, where adaptations come from and mindset towards SARDs.

### Participants

Interview participants were sampled from those who consented to be interviewed during our INSPIRE and Challenges in Care surveys. Eligible patients were 18 + , spoke English, and self-reported a diagnosis of a SARD on a clinic letter. Eligible clinicians interacted with patients with SARDs. This included a broad range of clinician specialities due to the multi-system nature of many SARDs and hence the involvement of multiple specialities in their care. Sample size was guided by informational power [25]. Interview data were monitored during data collection, assessing the depth, variation and relevance to the research question alongside diversity of the sample in terms of experiences and demographics. The research team iteratively assessed and discussed the data ability to answer the research question. Once data were deemed to be rich and sufficiently articulate to answer the research question and did not generate any new themes, data collection ceased.

### Procedure

Ethical approval was obtained through the Cambridge Psychology Research Committee: PRE 2022.027 (INSPIRE) and PRE.2024.062 (Challenges in Care). All data was kept in accordance with the General Data Protection Regulation and the Declaration of Helsinki. During this process, participants consented to the publication of de-identified quotes and informed about data management. Ethical approval was centrally obtained from the UK with permission for international online recruitment. All participants provided recorded verbal consent prior to interviews.

Interviewees were purposively sampled to ensure a variety of demographics and disease experiences were captured. After consent, participants were interviewed via online video-conferencing software, or telephone, from October 2024-March 2025. The interviews were semi-structured and guided by an interview schedule. Interviews were around 60 min for patients and 40 min for clinicians.

### Analysis

Audio recordings of the interviews were transcribed verbatim, with removal of any identifying information to preserve anonymity and stored according to participant identification number. Transcripts were imported into NVivo [26].

A critical realist approach was taken. Reflexive thematic analysis was conducted [27–30] which identified patterns of shared meaning across the data in order to generate themes. AT immersed themselves in the data, coded interview and open-ended survey responses line-by-line inductively and developed preliminary themes. This was done iteratively with annotations made. Preliminary themes were shared with the wider research team and refined. Themes were written into a report using illustrative quotes and again were presented to the wider research team, including patient and charity partners. This process was iterative until final themes were agreed. For example, patient partners changed the order of themes presented, edited wording to ensure discussions around acceptance were reflective of a variety of patient experiences and renamed the preliminary subtheme “challenges to adapting” to “costs and challenges to adapting”. To enhance trustworthiness, AT utilised reflexive memos throughout analysis. Additionally, the team, including patient partners, discussed interpretations of the data including negative cases. Researchers’ social positioning was seen as a resource; the research team reflected on their intersecting and overlapping identities and how this impacted interpretation of participant meaning in quotes [31]. This is explored more in the Supplementary Materials.

## Results

### Participants

Patient participants (n = 31) interviewed were predominately white (65%), and female (77%). They had a range of diseases and time since diagnosis (Table 1). Clinician participants (n = 28) were predominately male (64%) and were predominately rheumatologists (43%) (Table 2). Full demographics of survey participants are reported elsewhere [32].

**Table 1:** Patient demographics and diagnostic characteristics. Multiple primary: Participant selected more than one primary diagnosis

Characteristic	Patients (n = 31)	
	**Count**	**Percentage**
**Age**		31
18–29	2	6%
30–39	4	13%
40–49	9	29%
50–59	7	23%
60–69	3	10%
70 +	5	16%
Undisclosed	1	3%
**Gender**		
Female	24	77%
Male	7	23%
Non-binary	0	0%
Other/undisclosed	0	0%
**Disease**		
Multiple primary	5	16%
Myositis	1	3%
Polymyalgia Rheumatica (PMR)	2	6%
Rheumatoid Arthritis/ Inflammatory Arthritis (RA/IA)	4	13%
Sjögren’s	4	13%
SLE	11	37%
Systemic sclerosis	1	3%
Vasculitis	3	10%
**Ethnicity**		
Asian	4	13%
White	20	65%
Latin/Hispanic	4	13%
Black	3	10%
Other/undisclosed	0	0%
**Time since diagnosis**		
< 1 year	3	10%
1–2 yrs	4	13%
3–5 yrs	5	16%
6–9 yrs	5	16%
10 years +	4	13%
Undisclosed	10	32%

**Table 2:** Clinician demographics

Characteristic	Clinicians (n = 28)	
	Count	Percentage
**Speciality**		28
Rheumatologist	12	43%
GP	5	18%
Nurse	3	11%
Psychiatrist	3	11%
Nephrologist	1	4%
Neurologist	3	11%
Psychologist	1	4%
**Gender**		
Female	10	36%
Male	18	64%
Other/undisclosed	0	0%
**Location**		
UK	26	93%
Caribbean	1	4%
South America	1	4%

Qualitative data reflected a multitude of experiences. Some patients implemented small changes and lived similarly to pre-diagnosis. Others were more chronically unwell and enacted many adaptations. The first theme describes the *journey with acceptance and adapting*, exploring *finding contentment* and that *adapting and acceptance are dynamic and non-linear*. The second theme explores the *mechanisms for identifying and enacting adaptations* which included the *techniques used for managing life-changing symptoms, empowerment and control* and *costs and challenges to adapting* to living with SARDs. The final theme explored that *holistic support is beneficial but not prevalent*.

### Theme 1. Journey with acceptance and adapting.

Patients were at various stages on their acceptance and adapting journeys. Many were still in the process of coming to terms with the pervasive impact of symptoms on their lives, and the impact on their identity:

"I'm not the person I was, and I'm just someone who's hanging on all the time … not doing a very good job. It's not who I want to be." (PT01, PMR, 60-69)

Patients often expressed grief for the life they once had:

“It feels like a grieving process and you do feel like you've lost the old version of yourself and you just having to and you having to get through accepting a new version of yourself, that has a huge effect on your mental health.” (PT02, SLE, 30-39)

There was often a period of fighting or resisting adapting or acceptance, particularly when life with a SARD was incongruent with how they saw themselves:

“Carried on at 1000 miles an hour, because that’s the way I am. Trying to, what’s the word, trying to not accept it. To think I could continue the way I been doing before the diagnosis, before I got it, and seeing how far I could push myself and if anything, I was pushing myself even more. Erm, trying to beat it, if you like.” (PT03, SLE, 40-49)

This fighting against the disease was recognised as psychologically and physically demanding by patients: "*It's taking less like energy, not to fight it, I think. It's much easier now to just accept the things I can and cannot do*" (PT04, SLE, 30–39) and clinicians:

Often, an initial level of shame was expressed around adaptations and becoming less independent. However, the improved quality of life from accepting help was highlighted:

"People are offering to take me places and to help me in day-to-day things or just keep me company. And now that I've let them, rather than pushing everybody away and kind of just hiding, it's made a big difference." (PT04, SLE, 30-39)

Others discussed how they knew acceptance was beneficial but had not yet been able to engage with the process:

"Lots of the people on [online forum] say, oh, the secret is to just accepting your illness and coming to terms with it. And I agree with that. But I haven't managed it." (PT01, PMR, 60-69)

Although patients often referred directly to engaging with this process as “*acceptance*” (multiple participants), negative connotations and pressure were associated with this term.

“I don’t really like the word acceptance because it suggests the use of force onto someone which they have to accept…just feels to me like someone is pushing something on somebody” (CPT17, Neurologist)

Patients and clinicians often could not pinpoint how or why acceptance and adapting occurred, and that the process was highly individual.

A small minority of clinicians appeared to place blame on some patients for struggling with adapting by dismissing life-changing symptoms such as fatigue:

### Subtheme 1.1: Finding contentment.

Many patients described the process of adapting and accepting as difficult. Some, particularly those with longer-standing disease, described finding contentment over time. This often focused on finding the “*slivers of joy”* (PT05, SLE) in less physically and/or mentally demanding activities that could work within the constraints of debilitating symptoms such as fatigue:

“My quality of life has changed from being fit and very active to enjoying more sedentary activities and listening to my body when trying to push myself …It has been a big learning curve!” (PT06, multiple primary, 60-69)

Some patients and clinicians felt that achieving contentment was as a result of underlying beliefs, a “*get on with it*” (multiple patients) mindset and perspectives on illness or life: “*I just think it's I am an optimist and I enjoy life*” (PT07, Vasculitis, 60–69).

Most clinicians were empathetic towards the difficulties in adapting. They reflected the importance of finding joy in slower paced aspects of life such as “*walking, gardening… painting… singing*” (CPT01, Rheumatologist), and focusing on what could be achieved providing a sense of purpose and fulfillment:

“All you can do is look forwards and not say what you can’t do but think about what you can do and take pleasure in even the smallest things you can now do and build on that… it’s learning to help people find pleasure and reward.” (CPT03, Rheumatologist)

### Subtheme 1.2: Adapting and acceptance is dynamic and non-linear.

Most patients’ journeys with adapting and accepting living with SARDs were not linear. The enormity of this task, including confusion and fear with ever-changing symptoms was discussed. The unpredictability of symptoms, and diagnoses/misdiagnoses, meant regular readaptations: *“You're just getting used to it and something else comes along”* (PT01, PMR, 60–69), which impacted acceptance. Conversely, some patients reported adaptations were unnecessary when in a period of remission or lower disease activity.

Patients reflected on the awareness of the threat of flares even during periods of remission: *“You're doing well, but you've got to realise, it’s sneaky… you also can't rest on your laurels*” (PT08, vasculitis, 50–59). Some clinicians reported that some patients learnt to adapt their lives to the relapsing–remitting nature of SARDs whereas others reflected on the psychological impact of uncertainty and ever-present fear of relapse:

“I do have a number of patients who I think probably have post-traumatic stress disorder from often, from the time of diagnosis or from a major flare, and those patients sometimes can find it very difficult to adapt to having a chronic illness because they have a lot of anxiety and fear that this is going to come back.” (CPT04, Rheumatologist)

Some patients reported that adaptation strategies evolved over their disease course, which included patients learning to listen to their body:

“Nobody knows your body better than you do" (PT09, Sjögren’s, 70+).

This involved making daily decisions of required adaptations, rather than focusing on linear progress:

"Every day is different. So just deal with whatever you wake up to." (PT04, SLE, 30-39)

### Theme 2. Mechanisms for identifying and enacting adaptations

Patients described the process they engaged with to identify and carry out adaptations. Adaptations with anecdotal evidence were found on online forums such as Health Unlocked or support groups. These were used to gain “*tips and ideas”* (PT10, RA, 30–39) and to educate on the diseases:

“The charity forum has been my absolute gospel, if you go to how to manage it yourself.” (PT11, vasculitis/Giant Cell Arteritis , 70+)

Other patients were wary of applying others’ experiences of their condition to their own life as “*what works for you might not work someone else*” (PT08, vasculitis, 50–59). Several patients spoke about wanting adaptations to have scientific backing for them to be seen as an eligible candidate to try:

“Where's the paper to support your claim? You know, like is it published in a journal? Otherwise, I'm not listening to you.” (PT12, SLE, 40-49)

The lack of evidence and tendency for media sensationalism with regards to diet, resulted in patients and clinicians noting the importance of using logic and enacting things which “*make sense*” (PT13, multiple primary, 30–39) when trying adaptations. Experimentation using anecdotal evidence on diets (without being highly restrictive) was encouraged:

“I tell them to experiment, things like dietary modification. Very poorly science driven but if you want to say, cut out tomatoes, try a FODMAP or gluten free diet, then try it” (CPT05, Rheumatologist)

Most patients reported engaging in trial-and-error experimentation process to see if an adaptation worked for them. Although this was time-consuming, once positive adaptations were identified, some patients described them as becoming an integrated part of their day-to-day lives.

### Subtheme 2.1: Techniques for managing life-changing symptoms

Almost all patients discussed that fatigue was severely limiting in multiple domains and required conscious management. Adaptations included pacing, resting, engaging in light exercise, and organising schedules to ensure there were not too many activities in one day:

“I will do the washing this afternoon, I will write that difficult letter and perhaps one thing each day, and you work out the week in advance planning" (PT01, PMR, 60-69)

This often resulted in having to accept that some activities would not be completed or that assistance from others would be required. Pacing was considered inadequate for overcoming the fatigue, and induced frustration at the lack of alternative support:

“I'm always told that none of the drugs will help with fatigue, and yet this is far and away the worst symptom…‘Pacing' is not a treatment, it's a coping strategy that puts limits on your life.” (PT16, IA/RA, 50-59)

Patients often reported that when engaging in activities, they bargained with oneself as to how much energy to expend and the later consequences of this. The ‘spoon theory’ was discussed by some patients with regards to energy management:

“I have X number of spoons per day and I can't go, that number of spoons is probably less than the average person and so I need to be cautious about how many spoons I use.” (PT13, multiple primary, 30-39)

Stress was considered to worsen the disease, therefore patients avoided it where possible. Social support from friends and family was important, however, some did not have this:

“The ones who are struggling, single mothers, isolated people, people in difficult relationships, people with large families to manage, the more responsibility they have for others and the less personal support they get with their illness the more difficult it is to manage.” (CPT03, Rheumatologist).

Some patients adjusted their work in a variety of ways such as having flexible hours and working from home, taking time off work or leaving employment altogether.

Diet changes were also frequently discussed to reduce inflammation, as well as altering medications such as corticosteroids to help manage symptoms. Patients who were religious spoke about utilising faith and mindfulness to support themselves emotionally, including praying for how to live well with a SARD:

"I don't pray to get better. I never did that, but I just feel that in life, everybody has their destiny. And just pray for my for strength and to get me keep me going." (PT07, Vasculitis, 60-69)

Physical symptoms were also adapted to in a variety of ways such as making one’s house more accessible, using mobility aids or avoiding warm/cold environments:

"Things in the kitchen to help, like a water machine instead of using the kettle because I've had quite a few accidents of dropping the kettle." (PT04, SLE, 30-39)

### Subtheme 2.2: Regaining a sense of control

Increasing knowledge was viewed as key to feeling more in control:

“You give them some control to make them do things to feel better, it’s that knowledge and power.” (CPT06, Psychiatrist)

“I think trying to encourage people to take responsibility for their own illness and allow them to grow with that, rather than me doctor you patient, me telling you what to do… I think if you can empower patients to manage these illnesses and think about what they can manage to do, a huge part of acceptance and looking after people holistically and their mental health into the mix.” (PT19, GP)

Clinicians also reflected that feeling in control was important to adapting as this could empower patients, even if this was the illusion of control:

### Subtheme 2.3: Costs and challenges to adapting

Patients described a variety of challenges to enacting adaptations including feeling negatively about oneself and feeling guilt for carrying out adaptations:

“I have a lot of guilt. Really bad. So that sort of plays with me quite a bit.” (PT20, SLE, 40-49)

Many patients reported having to decline invitations to socialise or cancel at the last minute. This had a negative impact on their quality of life and relationships, inducing a fear that invitations would cease:

“I just never say, yes I’ll do anything, because what’s the point, you’re just going to let everyone down, aren’t you?” (PT21, RA, 60-69).

"I could just say "no, thank you to coffee" but … You don't want to make yourself too much of a pain, really. Because then of course people will stop inviting you" (PT01, PMR, 60-69).

While some patients reflected the need to renegotiate, or even lose relationships, others reported that “*that true friends will accept it*” (PT08, vasculitis, 50–59).

Social consequences were perceived to be as a result of others’ limited knowledge of SARDs. The invisible nature of symptoms meant others did not understand the need for adaptations, or “*people just forget because it’s not obvious*” (PT15, RA, 50–59) resulting in lack of empathy. Nebulous but debilitating symptoms meant some patients had to refuse social invitations due to them being incongruous with their necessary adaptations:

“I have very severe gastroparesis and have a liquid only diet which means I have had to say goodbye to meals out with friends and family. At first my family thought I was just making a fuss and attention seeking” (PT22, Sjögren’s, 70+)

Enacting adaptions in public and professional context had social consequences which caused patients to feel judged, uncomfortable or lonely. Some patients discussed pushing themselves to forego the adaptation to avoid upsetting others or being socially different.

Several patients also noted that socioeconomic status influenced whether adaptations could be enacted due to their costs:

“I feel I've adjusted well and I'm fortunate that my situation allows me to do only what I want to do and that I can afford to hire help” (PT24, Vasculitis, 70+)

Changing of working hours or having to retire also impacted finances. For those in countries without a free health system, the costs of clinics and medications were sometimes unaffordable: *“I'm still getting collection letters from tonnes of doctors, hospitals because, I can't afford to pay it*” (PT25, Scleroderma, 30’s). Regardless of free or paid healthcare, adaptations often involved financial costs for items such as sun protection, dental care and increased heating bills. This created disparities in the adaptations patients could enact.

### Theme 3: Holistic support is beneficial but not prevalent

Patients reported a very limited health service support in adapting to SARDs with the onus largely on the patient:

“I feel that the medical profession have pretty much left me to manage this condition on my own” (PT26, PMR, 60-69).

A minority of patients described the value of the holistic and multidisciplinary support they had received.

Rheumatologists lamented the lack of holistic support:

“It is not enough at all. We need psychologists in our multidisciplinary team for rheumatic diseases patients, it should be a rule, but we have not yet” (CPT07, Rheumatologist).

Most physicians reported that controlling the disease had to take priority. Time constraints were raised by all rheumatologists and GPs as limiting their ability to assist with adapting, regardless of country or health system.

Clinicians were often sympathetic about adapting challenges, with some emphasising the importance of validating patients’ experiences despite feeling ill-prepared:

“I just make it up as I go along, is the honest answer. There is no training for this whatsoever” (CPT08, Rheumatologist)

Some rheumatologists and GPs felt that helping patients adapt was not within their remit, or expertise, and would not be cost-effective as exemplified in Table 2. Rather, most rheumatologists saw their role to signpost and coordinate:

“The analogy of the conductor of the orchestra. Right? So, we don't play the instruments, but we sort of help patients to navigate different specialties or different health professionals and make sure everyone's talking to each other” (CPT04, Rheumatologist).

Rheumatologist nurses and allied health professionals were often viewed as having greater skills and time to include psychosocial aspects into their provision of care as exemplified in Table 3. Psychiatrists could also be important in supporting patients in adapting as they *“see patients not diseases”* (CPT18, Psychiatrist).

**Table 3:** Illustrative quotes of healthcare professionals perceived roles of psychosocial support

Description	Illustrative Quote
Rheumatologist and GP’s view helping with adapting as not within their remit or expertise	*This [clinic time] is not long enough. However, this is the only feasible option considering the load of patients [of] 250 patients with rheumatic diseases per clinic, and the manpower crunch. Social and psychological assessment is neglected the most” (*CPT07, Rheumatologist)
	“*Our principle here is prudent healthcare so I will see those with highest clinical need so they’re not being caused harm by us neglecting them…So from a medical point of view what is worse someone going into renal failure because you didn’t see them on time or someone feeling miserable because they’re tired and we’re not doing anything about it?”* (CPT02, Rheumatologist)
	*“It wouldn't be very cost effective paying a GP to do it [teach adapting], and I'm just thinking, when would we do it? We'd have to make a consultation for that and there are no consultations. There are no spaces for that”* (CPT09, General Practitioner)
	*“Just the same as GPs have limited appointment time, we have limited time. I think there is a role for specialist nurses, but the key is continuity of care and I think that's what patients value more and benefit from most is seeing the same person time after time after time”* (CPT10, Rheumatologist)
Rheumatologist nurses and allied health professionals as supporting patients with adapting	*“The rheumatologists are treating them as a disease, not as a person…a good rheumatology nurse can help biopsychosocially… A good occupational therapist can do wonderful jobs with pain and energy and pacing. It’s all about adapting to lives and a good clinician should always be looking at their sleep, their relationships…their coping, their lives”* (CPT11, neurologist)
	*It’s like a CBT based programme, so we do a whole session on sleep, we look at pacing, we look at erm, thoughts, communication skills, we look at stress and anxiety, we look at setbacks* (CPT12, nurse)
	*“It seems more acceptable to be more chatty with me…they’ll talk to me more easily about things they think the doctors won’t want to know about…the impression that my patients give me is that the doctors are too busy and a stage removed from them”* (CPT13, nurse) “*I think lupus nurse is a very important factor because lupus nurse can really give that extra dimension of care that doctors simply are incapable of giving due to time and their own personal tendencies. Because doctors basically give you medication at that's it, but lupus nurse can actually, might be able to give you advice. You know, rest more, you know*” (PT27, SLE, 60–69)
	*“I ask who they’re seeing, family support, we go through all of those, the fatigue, mood, so because I’m a nurse we go through that more…I do a lot of lifestyle management”* (CPT14, nurse)

Therapy could assist the process of adapting, by giving patients a space to share feelings with non-judgemental reactions and build on their self-esteem:

“I was talking to my therapist, and she sort of said “it’s grief”. I said “what?” And she said “you are grieving for the person that you were” and I thought oh my God and you’d never think of grief but I’m grieving for the person I used to be before I was sick.” (PT28, Sjögren’s, 50-59)

However, patients raised frustrations when therapists had limited knowledge and expertise with SARDs which could result in dangerous advice or an ineffective therapeutic relationship:

“He [therapist] certainly was not trained for somebody like me…he was really pushing my button in the wrong way” (PT29, SLE, 60-69).

Clinicians noted the need for more support on adapting, particularly at diagnosis and for tailored therapeutic approaches for this population:

“Just because it isn’t a mental health condition, doesn’t mean their emotional and mental health won’t be profoundly affected by the diagnosis you’ve just given them. I’d love to see a situation where all newly diagnosed SLE patients are automatically given a referral to work with a psychologist.” (CPT16, Psychiatrist)

## Discussion

Patient and clinician perspectives on adapting to living with SARDs highlighted three themes. The first explored adapting as a non-linear, ever-shifting journey, which for some involved acceptance. Second, patients used various methods to identify and implement adaptations, but these came with social and financial costs. Finally, holistic and psychological support was beneficial but rarely offered, with clinicians citing lack of time and training. This study presents novel insights into adapting that demonstrates multiple commonalities between all SARDs in the process and challenges of adapting.

Our results reflect previous research on rheumatic and musculoskeletal diseases, that adaptations are a crucial part of managing patients’ disease in daily life, and that was is enacted is dependent on feasibility and personal preference [33]. Our findings suggested patients utilise knowledge when adapting. A previous review found low health literacy in patients with chronic illnesses may impact behaviours needed for development of self-management skills [34]. A literature review of self-management approaches of people with chronic conditions highlighted the necessity for patients to select strategies which work for them as it is highly individual [35]. We found nurses and allied health professionals as crucial for increasing patients’ ability to adapt to living with a SARD. This is reflected in previous research whereby rheumatology nurses were found to be necessary to optimise patient outcomes and meet individual needs [36]. Thus, there is a need to equip such healthcare professionals with knowledge and resources to provide this crucial care [36]. Mirroring this and other literature [14], we found that patients utilised trial-and-error experimentation to identify adaptations that worked for them. In line with previous literature, we also found patients adapted by learning about their condition and using religion for support [13, 15, 19, 37].

Our findings indicate that patients experienced emotional distress from non-acceptance of their SARD, with acceptance providing relief. This aligns with research emphasising that adapting through accepting new bodily limitations and realities is crucial, and more effective than fighting the condition, despite requiring more time [18, 23, 38–40]. Some patients reflected adaptations were incongruent with how they saw themselves, mirroring self-discrepancy theory which has previously applied to mental health conditions [41]. However, our results imply it has relevance to SARD populations.

Young adults with autoimmune diseases have been reported to create a “new normal” when adapting [42], and our research extends this concept to apply across all ages. Disease acceptance, positive attitude, and learning to listen to ones body have all been identified as key components for adapting [18, 38, 39, 43]. Our study found some patients described a similar process with acceptance, including finding contentment. Nevertheless, it is important to note that acceptance is influenced by respective levels of disease activity and disability. Moreover, this process is more difficult when sufficient medical treatment is not provided. Our qualitative patterns suggest that irrespective of pre-disease personality, maintaining a positive mindset is challenging for those with permanent damage or inability to work/participate in daily life. A minority of clinician participants suggested difficulties in accepting and adapting were as a result of lacking the “correct” mindset. This perspective could increase stigma and self and societal blame.

Maintaining hope and optimism through social interaction and reaching goals has been identified as important to patients [19]. However, we found some patients struggled with acceptance, a challenge not consistently reflected in the existing literature. In our findings, patients and clinicians saw adapting as a non-linear process. This has been reflected in other research with systemic sclerosis patients reporting changing their strategies to adapt with their changing symptoms [14]. The Patient Health Engagement (PHE) model [44] describes four psychological experiences when engaging with healthcare including initial overwhelm, anxiety and knowledge acquisition, acceptance and holistic empowerment. [45] In our *journey with acceptance and adapting* theme, patients articulated a process akin to that described in the PHE model [44] whereby they moved from overwhelm (blackout) to anxiety (arousal) to acceptance (adhesion). The PHE model’s eudaimonic project stage is the final stage, however, we argue that due to SARDs’ fluctuating nature, patients rarely remain in this state.

Patients explicitly described living with a SARD as a process of grieving for life changes. This association is not commonly seen in literature, despite loss being acknowledged [46]. We found the five-stage grief model [47] was observed in patients experiences, specifically with some expressing anger, depression and acceptance. Unlike grief models that are often based on a singular event (e.g. death), SARD populations experience a changing landscape which induces grief. Subsequently, acceptance is non-linear and cannot be ‘obtained’. Instead, it is an oscillating process where individuals can move between the phases at any given time. This model is helpful for naming and validating the emotional experiences patients may go through. However, the Tonkin model [48] whereby one’s life grows around the grief is also a helpful complimentary visualisation for patients’ dynamic experiences. Social support, including from peers, family and organisations, is recognised as a coping mechanism for pain [14, 40]. A scoping review found inflammatory arthritis patients’ significant others could have a positive and negative impact on their adapting abilities, emphasising the need to support patients’ support network [49]. Our study also found social situation could have negative ramifications. We also found social support was important in identifying and enacting adaptations.

Participants called for tailored, evidence-based psychological interventions to support adapting to living with a SARD to be offered. Previous research has found some established therapies are effective in reducing psychological distress and improving quality of life in SLE patients [50, 51]. However, no study to date compares the efficacy of therapies in SARD populations. However, innovative interventions to support patients’ wellbeing are being trialled[52]. Other researchers have also called for a need for psychosocial interventions such as patient support groups, educational materials and peer counselling [53]. Offering psychosocial support could have expansive impacts, including empowering patients to have difficult conversations about their SARD.

We found that fatigue and pain were common targets of adaptations, often in substitution of them being optimally medically treated. While some patients achieved disease control with medication, many still experienced life-changing symptoms despite extensive treatment. This highlights the need for psychosocial support for all SARD patients in addition to pharmacotherapy.

### Strengths and limitations

A strength of this study is that the adaptations discussed in interviews were patient-led. It is also strengthened by the inclusion of a diverse range of both clinician and patient perspectives. However, young and male patients are underrepresented in the sample and therefore results may not be transferrable to these populations. The research is limited by not including patients who do not have the technological skills, ability or digital access to complete an online survey or to take part in remote interviews. Further research should investigate the impact of sociodemographic differences quantitatively. All diagnoses were self-reported and not verified with medical records.

### Future research and clinical implications

In agreement with other researchers [53], our findings identified the need for an evidence base for tailored psychological interventions to support adapting to living with a SARD. Services should offer psychological interventions such as counselling or grief support groups. However, further research is required to compare the effectiveness of evidence-based psychological interventions in supporting SARD populations adapting, such as Cognitive-Behavioural Therapy and Acceptance and Commitment Therapy. Previous research has trained rheumatology clinicians to support patient adapting, and they reported that cognitive behavioural and communication skills enhanced their practice [54]. Subsequently, authors call for regular skills-based rheumatology training to be delivered alongside clinical supervision [54]. Further research, including implementation studies, are required to determine how best to embed this in practice, including how t services can allow for time for clinicians to discuss adapting and proactive signposting to services.

Due to the limited transferability of results to men with SARDs, future qualitative research should focus on the experiences of men living with SARDs as there is a lack of research available [55]. Furthermore, an evidence base of beneficial/harmful adaptations for specific conditions and for different sociodemographic characteristics is needed, particularly for developing targeted and equitable health policies.

Medications were developed to limit organ involvement and increase life expectancy which has been the focus of many previous clinical trials with the neglect of including measurement of the efficacy on patients’ quality of life/functionality when using these treatments. Subsequently, patient-reported adaptations were necessary to ensure some functionality/quality of life. Furthermore, there is a therapeutic gap as SARD patients’ fatigue is pervasive yet often inadequately treated due to medications often being ineffective for fatigue. This resulted in almost all patients using pacing rather than an effective treatment. Future research clinical trials should look at treatment of fatigue as an urgent priority.

In conclusion, this study highlights the complex, individualised, and non-linear nature of adaptation to living with a SARD, emphasizing the role of social support, trial-and-error experimentation, and the challenges of (not) reaching acceptance. Future research should explore how best to support patients in the process of adapting to living with a SARD, ensuring that healthcare services provide holistic, tailored interventions to improve both physical and psychological well-being.

## Supplementary Information

Below is the link to the electronic supplementary material.

Supplementary file1 (DOCX 14 KB)

## Data Availability

Anonymised data may be available on reasonable request to the corresponding author.
