# Supplementary material for: Adapting to living with systemic autoimmune rheumatic diseases; a qualitative exploration of patient and clinician perspectives
Source: Rheumatol Int. 2025 Dec 15;46(1):10. doi: 10.1007/s00296-025-06033-9 (PMC12705830; doi:10.1007/s00296-025-06033-9)
Supplement: Supplementary file 1 — Supplementary file1 (DOCX 14 KB) [file 296_2025_6033_MOESM1_ESM.docx]

## Supplementary Materials

### Reflexivity

The interviewers took time to reflect on their interviewer role by allowing the interview to be a welcoming space for participants to share their experiences and be respectfully listened to, while being mindful she was not providing a signposting/referral/therapy session. Additionally, the authors involved in analysis tried to have awareness of their pre-existing attitude that it is more beneficial to adapt to living with a SARD rather than not accepting it. Finally, the core-research team were comprised of both men and women with a range of ethnicities and ages, with multiple team members having lived-experience of a SARD. Additionally, clinicians from a variety of fields contributed to the project. Thus, the development of themes and subthemes was impacted through these lenses.
